# Complete Genome Sequencing of *Streptomyces* sp. Strain MOE7, Which Produces an Extracellular Polysaccharide with Antioxidant and Antitumor Activities

**DOI:** 10.1128/genomeA.00442-17

**Published:** 2017-06-01

**Authors:** Marwa O. Elnahas, Kara B. De León, Magdy A. Amin, Mohamed M. D. Hussein, Amal E. Ali, Judy D. Wall

**Affiliations:** a Department of Biochemistry, University of Missouri, Columbia, Missouri, USA; b Department of Microbiology and Immunology, Faculty of Pharmacy, Cairo University, Cairo, Egypt; c Department of Chemistry of Natural and Microbial Products, National Research Center, Cairo, Egypt

## Abstract

*Streptomyces* sp. strain MOE7 is a Gram-positive filamentous bacterium isolated from agricultural soil in Columbia, Missouri, USA. Strain MOE7 produces an extracellular polysaccharide with antioxidant and antitumor activities. Through PacBio RSII sequencing, the MOE7 genome was found to be a linear chromosome of 8,399,509 bp with 6,782 protein-coding sequences.

## GENOME ANNOUNCEMENT

Members of the *Streptomyces* genus are Gram-positive filamentous bacteria that are mainly found in soil (1). Sequencing the genomes of *Streptomyces* is of great interest due to the commercial importance of their biologically active compounds (2). Due to their unique structures and pharmacological activities (3), extracellular polysaccharides are targeted as biological macromolecules of interest. Polysaccharides from *Streptomyces* species are not extensively studied compared to other bacterial sources. *Streptomyces* sp. strain MOE7 produces an extracellular polysaccharide with antioxidant and antitumor activities (M. O. Elnahas, M. A. Amin, M. M. D. Hussein, A. E. Ali, and J. D. Wall, unpublished data).

*Streptomyces* sp. MOE7 was isolated from an agricultural soil sample on ISP2 medium (4). For routine culturing and DNA isolation, strain MOE7 was grown in a medium containing, per liter: 5 g of tryptone, 5 g of yeast extract, 10 g of glucose, 3 g of anhydrous K_2_HPO_4_, 1 g of KH_2_PO_4_, 3 g of NaCl, 0.5 g of MgSO_4_·7H_2_O, and 0.5 g of anhydrous CaCO_3_ (5) at pH 7.0 with shaking at 180 rpm and 30°C for 2 days.

For genome sequencing, DNA was isolated with a Promega Wizard genomic DNA purification kit (Promega Corp., Madison, WI, USA), according to the manufacturer’s instructions, and was sequenced by PacBio RSII at the University of Maryland Institute for Genome Sciences. Sequences from two single-molecule real-time cells (6) were assembled with the Celera Assembler (7) into one contig with an average coverage of 180×. The analysis showed that the whole genome consists of a linear chromosome (8, 9) of 8,399,509 bp, with a centrally located replication origin and high G+C content of 72.0%.

The genome was annotated by NCBI Prokaryotic Genome Annotation Pipeline (10), which identified 6,782 protein-coding sequences and 67 tRNA, 21 (seven 5S, seven 16S, and seven 23S) rRNA, and three noncoding RNA (ncRNA) genes. The terminal regions of the chromosome showed an inverted repeat (9, 11) of 19,413 bp. Alignment of these regions revealed that the inverted repeats had only one mismatch and 18 insertions or deletions. The chromosome contained an additional 87,793 bp at the 5′ end that were not present in the 3′ inverted repeat. Coverage of this extension was approximately twice that of the rest of the chromosome, suggesting that it is either a plasmid with two copies per chromosome, or that it is also present at the 3′ end but was not distinguishable in the assembly process. PCR and sequencing across the junction of this extension and the inverted repeat region confirmed that these sequences are joined and the assembly was not in error. These ca. 88 kb are likely an extension of the inverted repeat present at both ends. Adding this sequence to the 3′ end makes the chromosome total length 8.5 Mb.

This genome sequencing provides the potential to improve the production of the polysaccharide as well as to discover other secondary metabolites produced by strain MOE7.

### Accession number(s).

This whole-genome shotgun project has been deposited in DDBJ/ENA/GenBank under the accession no. CP019779. The version described in this paper is the first version, CP019779.1.
